# Prospective Comparison of QFT-GIT and T-SPOT.TB Assays for Diagnosis of Active Tuberculosis

**DOI:** 10.1038/s41598-018-24285-3

**Published:** 2018-04-12

**Authors:** Fengjiao Du, Li Xie, Yonghong Zhang, Fei Gao, Huibin Zhang, Wei Chen, Bingqi Sun, Wei Sha, Yong Fang, Hongyan Jia, Aiying Xing, Boping Du, Li Zheng, Mengqiu Gao, Zongde Zhang

**Affiliations:** 1 Beijing Key Laboratory for Drug Resistance Tuberculosis Research, Beijing Chest Hospital, Capital Medical University, Beijing Tuberculosis and Thoracic Tumor Research Institute, Beijing, 101149 China; 2 Tuberculosis Department, Beijing Chest Hospital, Capital Medical University, Beijing Tuberculosis and Thoracic Tumor Research Institute, Beijing, 101149 China; 3 Department of Cardiology, General Hospital of Chinese People’s Armed Police Forces, Beijing, 100039 China; 4 Tuberculosis Department, Inner Mongolia Fourth Hospital, Hohhot, 010020 China; 5 Tuberculosis Laboratory, Shenyang Chest Hospital, Shenyang, 110044 China; 6 Tuberculosis Department, Pulmonary Hospital, Tongji University, Shanghai, 200030 China

## Abstract

T-SPOT.TB and QuantiFERON-TB Gold In-Tube (QFT-GIT) tests, as two commercial blood assays for diagnosing active tuberculosis (ATB), are not yet fully validated. Especially, there are no reports on comparing the efficacy between the two tests in the same population in China. A multicenter, prospective comparison study was undertaken at four hospitals specializing in pulmonary diseases. A total of 746 suspected pulmonary TB were enrolled and categorized, including 185 confirmed TB, 298 probable TB and 263 non-TB. Of 32 patients with indeterminate test results (ITRs), age and underlying disease were associated with the rate of ITRs. Furthermore, the rate of ITRs determined by T-SPOT.TB was lower than QFT-GIT (0.4% vs. 4.3%, *P* < 0.01). When excluding ITRs, the sensitivities of T-SPOT.TB and QFT-GIT were 85.2% and 84.8%, and specificities of 63.4% and 60.5%, respectively in the diagnosis of ATB. The two assays have an overall agreement of 92.3%, but exhibited a poor linear correlation (r^2^ = 0.086) between the levels of interferon-γ release detected by the different assays. Although having some heterogeneity in detecting interferon-γ release, both the QFT-GIT and T-SPOT.TB demonstrated high concordance in diagnosing ATB. However, neither of them showed suitability in the definitive diagnosis of the disease.

## Introduction

Tuberculosis (TB) is an airborne-transmitted infectious disease with high morbidity and mortality. In 2015, there were an estimated 10.4 million new TB cases worldwide. China is ranked third, worldwide (after India and Indonesia), with 918 thousand new cases of TB every year^1–3^. Early and accurate diagnosis of active TB is critical to the care of TB patients and to control transmission in these high-burden developing countries. Despite incorporation of clinical, radiological, pathological and microbiological examinations, TB diagnosis can still be difficult. Conclusive diagnostic tests, including microbial culture and smear for acid-fast bacilli, are not sensitive enough to identify all the active cases^4^.

Recently, interferon-γ release assays (IGRAs) have emerged as immunodiagnostic tools to detect tuberculous infection. IGRAs quantify interferon-γ released by T-lymphocytes in response to stimulation by specific antigens encoded in region of difference 1 (RD1) of the *Mycobacterium tuberculosis* (MTB) genome. Two IGRAs, including an enzyme-linked immunospot (ELISPOT) assay T-SPOT.TB blood test (T-SPOT.TB; Oxford Immunotec Limited, United Kingdom) and an enzyme-linked immunosorbent assay (ELISA) QuantiFERON-TB Gold In-Tube test (QFT-GIT; Cellestis Limited, Australia), are now commercially available. Several studies have demonstrated that IGRAs may be useful as supplemental tools in the diagnosis of active TB^5–8^. Since IGRA methods, interpretation criteria, and study populations varied considerably among published reports, it remains uncertain whether IGRA is suitable for diagnosing active TB or which IGRA is more effective in diagnosing active TB. Relatively few studies have reported direct comparison between T-SPOT.TB and QFT-GIT assays in diagnosing active TB, most studies being performed with either the T-SPOT.TB or QFT-GIT assay^9–12^. Furthermore, indeterminate test results (ITRs) of IGRAs could confuse clinical interpretation. If a sample does not respond sufficiently to either specific antigens or the mitogen control, the test result of this sample is deemed as an ITR, yet some evaluations included ITRs in their sensitivity calculations and limited information is available on the risk factors associated with ITRs^13–15^.

The aim of this study was to assess the diagnostic performance of QFT-GIT and T-SPOT.TB assays in patients with active TB, and compare the concordance between the two diagnostic assays in routine clinical practice in a high TB setting.

## Results

### Clinical characteristics of participants

**Figure 1:**
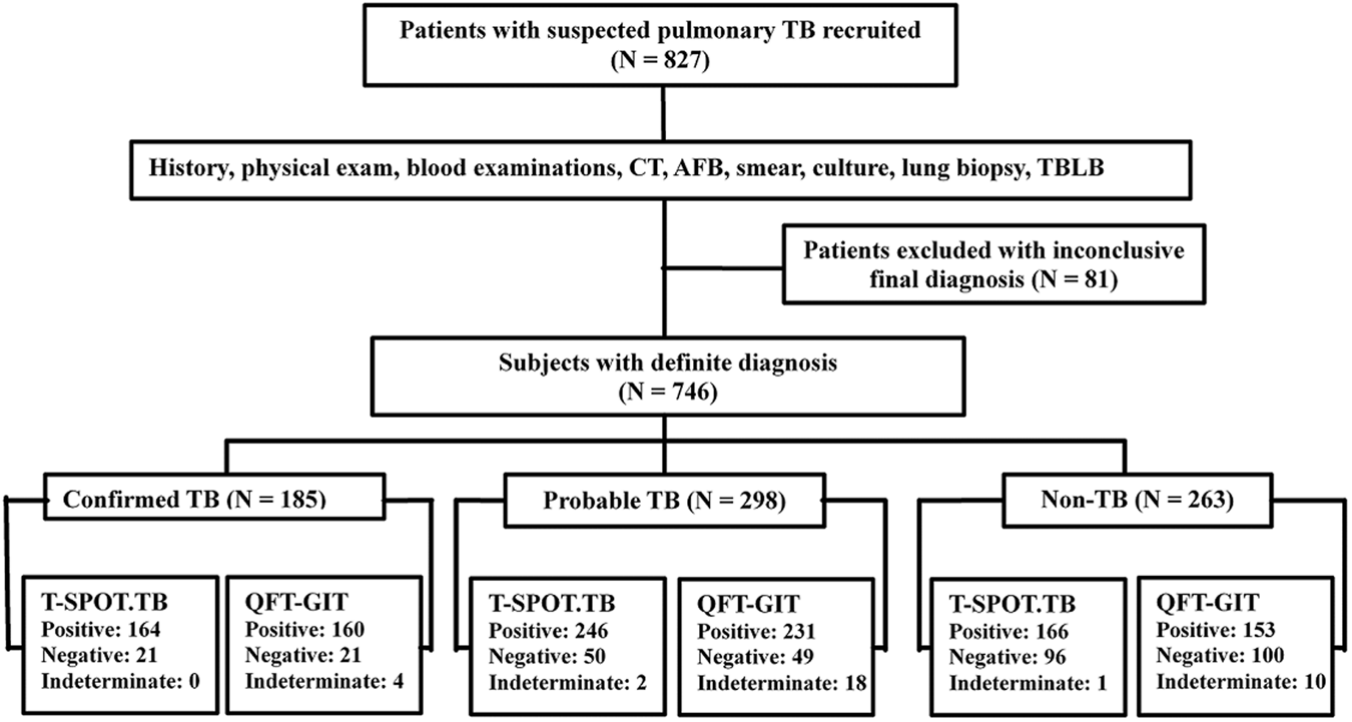
Flow chart of the study population. Of the 827 patients with suspected pulmonary tuberculosis recruited, 746 were eligible for inclusion in the final analysis. TB, tuberculosis. CT, computed tomography. AFB, acid-fast bacilli. TBLB, transbronchial lung biopsy.

In total, 827 participants with clinically suspected pulmonary TB were enrolled in this study. After a follow-up of at least 3 months, 81 patients were excluded from the study, 9 due to lack of data and 72 with no final diagnosis. The remaining 746 participants were ultimately included for T-SPOT.TB and QFT-GIT analyses (Fig. 1). All patients in the study were tested for HIV and all had negative results. Demographic and clinical characteristics of the patients are shown in Table 1. Based on the reference standard, 185 (24.8%) patients were categorized as having confirmed TB, 298 (39.9%) patients were categorized as having probable TB, and 263 (35.2%) patients were categorized as having non-TB. Other organs, in addition to the lung, were affected in 109 (14.6%) of the patients with TB, including lymph node, pleural membrane, bronchus chest wall and bone. Of the 263 patients in the non-TB group, 168 had lung carcinomas, 53 had bacterial pneumonia or a lung abscess, 13 had bronchiectasis and 29 had other lung diseases (Table 1).

**Table 1:** Clinical characteristics in study groups (N = 746).

Characteristics	Confirmed TB (N = 185)	Probable TB (N = 298)	Non-TB (N = 263)
Age, years, mean (range)	45 (19–79)	47 (23–81)	51 (26–84)
Male sex	126	179	161
Duration of symptom (days)	60 (30–150)	60 (30–180)	65 (30–100)
BCG vaccinated (based on presence of scar and vaccination records)	127 (68.6)	211 (70.8)	195 (74.1)
Underlying disease			
Diabetes mellitus	41	29	27
COPD	23	24	20
Connective tissue disease	3	2	5
Solid tumor	4	3	0
Virus hepatitis or cirrhosis	6	7	4
Intestinal obstruction	2	5	0
Hypoproteinemia	5	12	7

TB, tuberculosis; COPD, chronic obstructive pulmonary disease.

### Distribution of indeterminate IGRA results and identification of risk factors

Among the 746 TB patients, the T-SPOT.TB assay provided significantly fewer ITRs (0.4% [n = 3]) than did the QFT-GIT assay (4.3% [n = 32], Chi-square value = 24.6, *P* < 0.001). All 3 patients with ITRs determined by the T-SPOT.TB assay had positive control counts of <20 spot-forming cells (SFCs), while the 32 patients with ITRs determined by the QFT-GIT assay had mitogen minus Nil values of <0.5 IU/mL and antigen minus Nil values of <0.35 IU/mL. For these 32 patients, the T-SPOT.TB assay reported 3 indeterminate, 10 negative and 19 positive results. The 3 patients with ITRs determined by both the QFT-GIT and T-SPOT.TB assays were all greater than 50 years old and two of them had diabetes.

We further compared the basal characteristics and clinical laboratory findings for patients with indeterminate and determinate IGRA results (Table 2). Univariate analysis showed that age (*P* = 0.010) and underlying disease (*P* = 0.002) were significantly associated with ITRs. In multivariate analysis, older age [odds ratio (OR) = 2.84, 95% confidence interval (CI) = 1.34–6.02], and underlying disease (OR = 3.40, 95% CI = 1.65–6.99) were independent risk factors for ITRs.

**Table 2:** Univariate and multivariate analysis of risk factors associated with indeterminate IGRA results in 746 patients.

	Indeterminate N = 32 (%)	Determinate N = 714 (%)	*P* value^a^	Multivariate analysis	
				OR	95% CI
Age ≥ median age^b^	21 (65.2)	303 (42.4)	**0.010**	**2.841**	**1.339–6.024**
Male sex	18 (56.3)	448 (62.7)	0.458		
Duration of symptom (days)	63 (30–170)	61 (30–180)	0.927		
Underlying disease	18 (56.3)	208 (29.1)	**0.002**	**3.401**	**1.645–6.993**
Diabetes mellitus	7 (21.9)	87 (12.2)	0.106		
COPD	4 (12.5)	63 (8.8)	0.477		
Connective tissue disease	1 (3.1)	9 (1.3)	0.375		
Solid tumor	1 (3.1)	6 (0.8)	0.190		
Virus hepatitis or cirrhosis	2 (6.3)	15 (2.1)	0.124		
Intestinal obstruction	1 (3.1)	6 (0.8)	0.190		
Hypoproteinemia	2 (6.25)	22 (3.1)	0.275		
Microbiological findings					
Smear positive	4 (12.5)	181 (25.4)	0.100		
Smear negative	18 (56.3)	280 (39.2)	0.054		
Extra-pulmonary TB	8 (25.0)	101 (14.1)	0.089		
Laboratory findings					
WBC (10^3^/μL)	3876 ± 497	4156 ± 512	0.171		
Lymphocytes (10^3^/mL)	972 ± 54	1175 ± 63	0.136		
Total protein (g/dL)	3.32 ± 0.57	3.56 ± 0.71	0.412		
Albumin (g/dL)	2.87 ± 0.44	3.13 ± 0.51	0.534		
CRP (mg/dL)	8.28 ± 7.16	7.04 ± 8.12	0.219		

IGRAs, interferon-γ release assays. WBC, white blood cells. RBC, red blood cells. ADA, adenosine deaminase. CRP, C-reactive protein. ESR, erythrocyte sedimentation rate. ^a^*P* values in univariate analysis. Category variables were calculated by means of Chi-square tests or Fisher’s exact test, while continuous variables were calculated using the Mann-Whitney U test. *P* < 0.05 was the criterion for statistical significance and emphasized in bold. ^b^Median age^,^ 47 years old.

### Diagnostic performance of the T-SPOT.TB and QFT-GIT assays for active TB

Of the 743 valid results determined by the T-SPOT.TB assay, the sensitivities were 88.6% (95% CI = 83.3–92.5%) and 83.1% (95% CI = 78.4–87.0%) in the confirmed TB and probable TB groups, respectively, with no significant difference between the two subgroups (*P* > 0.05). The overall specificity was 63.4% (95% CI = 57.4–69.0%) in non-TB group (Table 3). The PPV, NPV, LR+ and LR− of T-SPOT.TB were 81.1% (95% CI = 74.3–87.8%), 70.3% (95% CI = 63.6–77.1%), 2.33 (95% CI = 1.98–2.74), and 0.233 (95% CI = 0.184–0.294) (Table 4).

**Table 3:** The sensitivity and specificity of the T-SPOT.TB and QFT-GIT assays in determining patients with active TB.

Groups	Methods	Number	Sensitivity % (95% CI)	Specificity % (95% CI)
Confirmed TB	T-SPOT.TB	185	88.6 (83.3–92.5)	
	QFT-GIT	181	88.4 (82.9–92.3)	
Probable TB	T-SPOT.TB	296	83.1 (78.4–87.0)	
	QFT-GIT	280	82.5 (77.6–86.5)	
Non-TB	T-SPOT.TB	262		63.4 (57.4–69.0)
	QFT-GIT	253		60.5 (54.3–66.3)
Total	T-SPOT.TB	743	85.2 (81.8–88.1)	63.4 (57.4–69.0)
	QFT-GIT	714	84.8 (81.2–87.8)	60.5 (54.3–66.3)
	Both assays^a^	714	87.5 (83.1–90.9)	58.5 (52.3–64.4)

TB, tuberculosis. ^a^A positive result was assumed when either test was positive and a negative result was assumed when both tests were negative.

**Table 4:** Diagnostic performance of the T-SPOT.TB and QFT-GIT assays in active TB.

Methods	Number	Sensitivity % (95% CI)	Specificity % (95% CI)	PPV % (95% CI)	NPV % (95% CI)	LR+ (95% CI)	LR− (95% CI)	Diagnostic odds ratio
T-SPOT.TB*	743	85.2	63.4	81.1	70.3	2.33	0.233	9.985
		(82.0–88.3)	(57.4–69.0)	(74.3–87.8)	(63.6–77.1)	(1.98–2.74)	(0.184–0.294)	(7.00–14.3)
QFT-G IT^#^	714	84.8	60.5	79.6	68.6	2.15	0.251	8.55
		(81.2–87.8)	(54.3–66.3)	(72.9–86.4)	(61.8–75.4)	(1.83–2.51)	(0.198–0.318)	(5.97–12.2)

TB, tuberculosis. PPV, positive predictive value; NPV, negative predictive value; LR+, likelihood ratio for positive test; LR−, likelihood ratio for negative value. *There were 3 indeterminate T-SPOT.TB results. ^#^There were 32 indeterminate QFT-GIT results.

Among 714 patients with valid QFT-GIT results, the sensitivities were 88.4% (95% CI = 82.9–92.3%) and 82.5% (95% CI = 77.6–86.5%) in the confirmed TB and probable TB groups, respectively, with no significant difference between the two subgroups (*P* > 0.05). The overall specificity was 60.5% (95% CI = 54.3–66.3%) in non-TB group (Table 3). The PPV, NPV, LR+ and LR− of QFT-GIT were 79.6% (95% CI = 72.9–86.4%), 68.6% (95% CI = 61.8–75.4%), 2.15 (95% CI = 1.83–2.51) and 0.251 (95% CI = 0.20–0.32) (Table 4).

Both sensitivity and specificity of T-SPOT.TB were slightly higher than those of QFT-GIT, but not reaching statistically significance (all *P* values > 0.05). We further measured the concordance between the tests using the Kappa index (k value > 0.75, excellent agreement; 0.75 ≥ k ≥ 0.4, fair to good agreement; k < 0.4, poor agreement). In the 714 subjects (excluding the 32 patients with indeterminate results by either of the two assays), both QFT-GIT and T-SPOT.TB were positive in 461 and negative in 197 subjects, and the observed agreement between the tests was 92.3%, with excellent concordance (k = 0.82) (Table 5). When investigating different groups, the agreements were 93.4%, 90.0%, and 93.7% in confirmed TB, probable TB and non-TB groups, respectively.

**Table 5:** Concordance between the T-SPOT.TB and QFT-GIT assays.

Groups	Number			T-SPOT.TB		Agreement (95% CI)	OR (95% CI)	Kappa
				Positive	Negative			
Confirmed TB	181	QFT-GIT	Positive	155	5	93.4 (86.6–100)	62.0 (17.4–221.1)	0.66
			Negative	7	14			
Probable TB	280	QFT-GIT	Positive	217	14	90.0 (83.2–98.8)	38.8 (17.0–88.2)	0.65
			Negative	14	35			
Non-TB	253	QFT-GIT	Positive	89	11	93.7 (86.9–100)	239.5 (80.6–711.8)	0.87
			Negative	5	148			
Total	714*	QFT-GIT	Positive	461	30	92.3 (85.5–99.1)	116.4 (67.1–202.0)	0.82
			Negative	26	197			

^*^Excluding 32 subjects with indeterminate results by either of the two interferon-γ release assays (T-SPOT.TB and QFT-GIT).

When combining the T-SPOT.TB and QFT-GIT assays in patients with probable TB, where a positive result was assumed when either test was positive and a negative result was assumed when both tests were negative, the diagnostic sensitivity increased to 87.5% (245/280), the specificity was 58.5% (148/253).

### Comparison of interferon-gamma concentration by IGRAs

**Figure 2:**
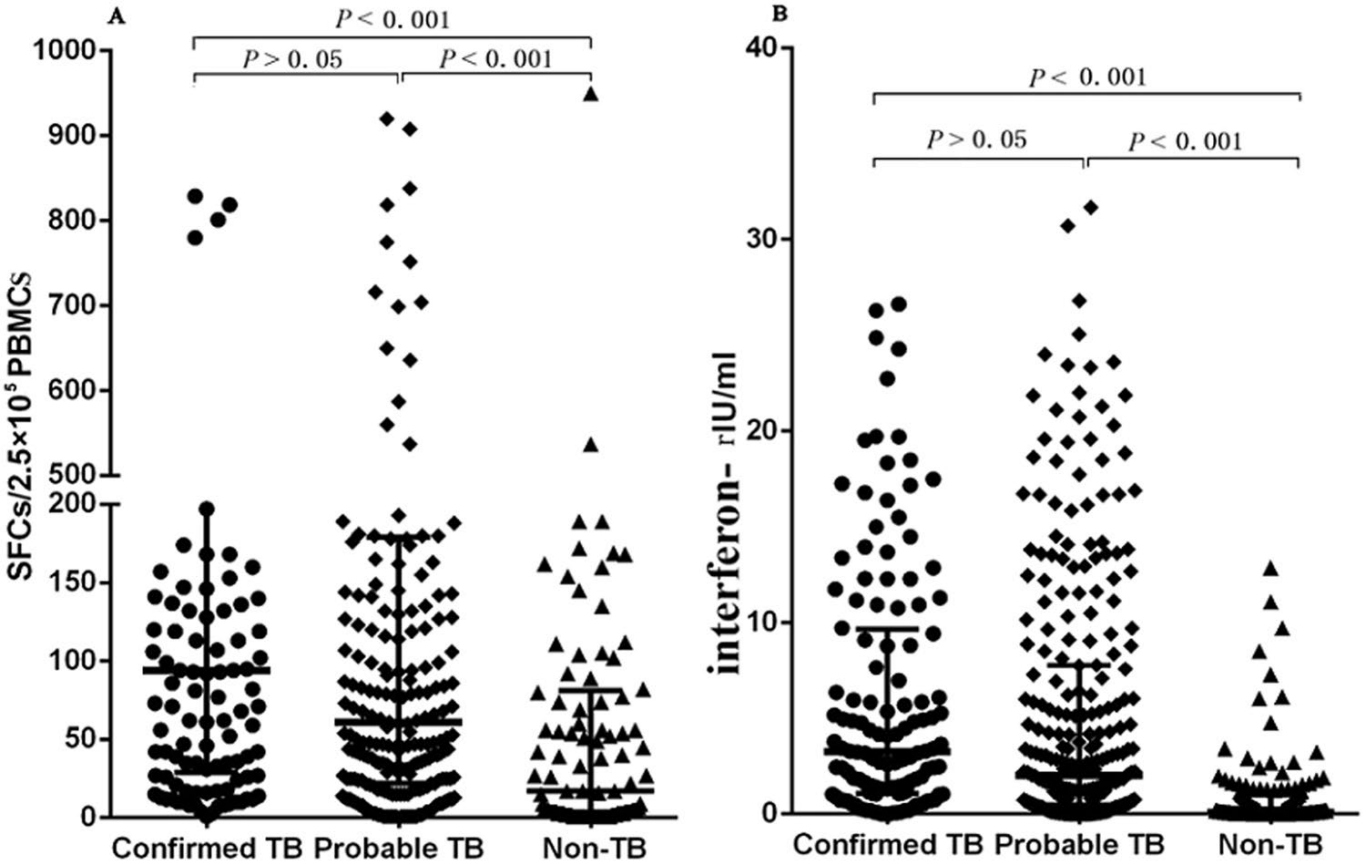
Scatter plots of the SFCs using the T-SPOT.TB assay (**A**) and the amounts of released interferon-γ using the QFT-GIT (**B**) assay in the confirmed TB, probable TB and non-TB groups, respectively. The groups were compared using Mann-Whitney tests. SFCs, spot forming cells. PBMCs, peripheral blood mononuclear cells. TB, tuberculosis.

The numbers of spot forming cells (SFCs) of interferon-γ determined by the T-SPOT.TB assay is shown in Fig. 2. Among 743 patients with valid T-SPOT.TB results, the median numbers of SFCs in the confirmed TB, probable TB and non-TB groups were 89 [Interquartile range (IQR): 22–213], 49 (IQR: 15–164) and 3 (IQR: 0–54) per 2.5 × 10^5^ PBMCs, respectively. Both the confirmed TB and probable TB groups had significantly more SFCs than the non-TB group (all *P* values < 0.001). The median numbers of SFCs in the confirmed TB group was greater than that in the probable TB group, but the difference did not reach statistical significance (*P* > 0.05).

Among the 714 patients with valid QFT-GIT results, the median concentration of interferon-γ in the confirmed TB, probable TB and non-TB groups were 3.46 (IQR: 1.17–10.5), 2.46 (IQR: 0.59–9.38), and 0.12 (IQR: 0.0003–0.910) IU/mL, respectively. Both the confirmed TB and probable TB groups had significantly higher interferon-γ concentration than the non-TB group (all *P* values < 0.001). Furthermore, the median interferon-γ concentration in the confirmed TB group was higher than that in the probable TB group, but the difference did not reach statistical significance (*P* > 0.05).

**Figure 3:**
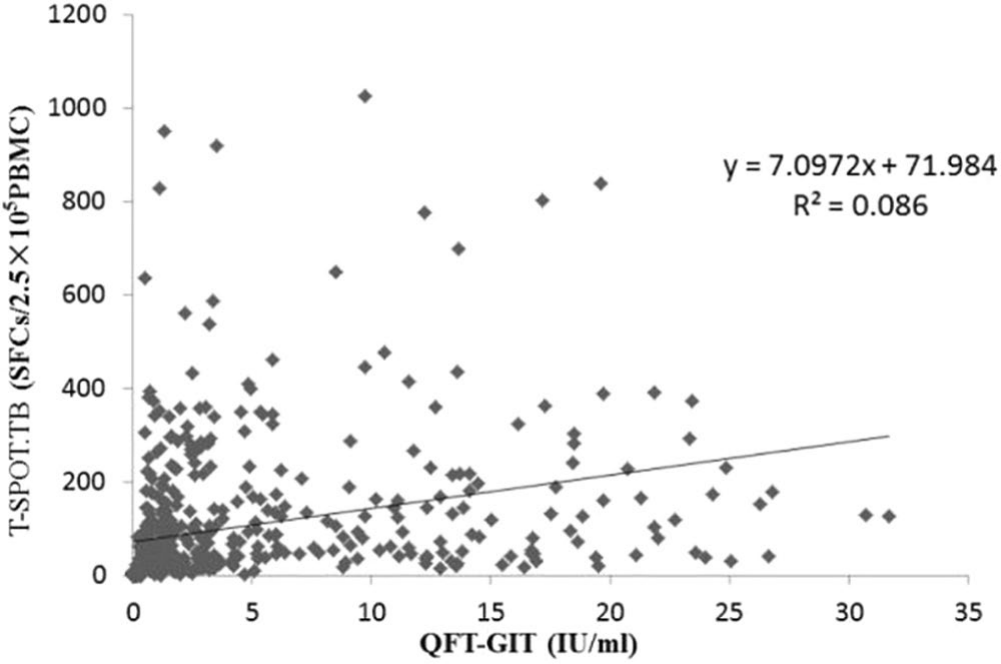
Association between the number of SFCs (spot-forming cells) (TB Ag – Nil) in the T-SPOT.TB assay and the amounts of released interferon-γ (TB Ag – Nil) in the QFT-GIT assay among 746 patients. Regression analysis were demonstrated by linear correlation (r^2^).

We further investigated the association between the number of SFCs in the T SPOT.TB and the amounts of released interferon-γ (measured by QFT-GIT). There was an increase of numbers of SFCs when interferon-γ concentrations became higher, with the regression line slope being 7.0972 (*P* < 0.001). However, regression analysis demonstrated a poor linear correlation (r^2^ = 0.086) between the two tests (Fig. 3).

## Discussion

Our findings indicate that, the two IGRAs (T-SPOT.TB and QFT-GIT) with different technical and performance characteristics might have some heterogeneity when used in routine clinical practice, although good agreement in general was observed between the two assays.

To our knowledge, this is the first study to date to directly compare the T-SPOT.TB and QFT-GIT assays for the diagnosis of active TB using the same study participants, in China. In our study, the overall sensitivity of the T-SPOT.TB assay was very close to that of the QFT-GIT assay, within the range from 60% to 89%^7,8,16–19^.And parallel with a recent meta-analysis, was 84.0% (95% CI 81.4%–86.4%) for T-SPOT.TB and 84.2% (95% CI 81.1%–87.0%) for QFT-GIT^20^. However, the sensitivity of T-SPOT.TB (85.2%) in our study was lower than the few studies performed in China by Zhang *et al*. (94.7%)^7^, Feng *et al*. (94.7%)^21^, and Kang *et al*. (93.2%)^22^. From the ten reported studies evaluating T-SPOT.TB in China, the combined sensitivity was 88%^23^. The sensitivity of QFT-GIT (84.8%) in our study was higher than the reported range in the earlier studies from highly endemic countries, Xia *et al*. (80.9%) in China^8^, 81% in India^24^, and 64.0% in Gambia^25^.

This multicenter, prospective study was conducted at four hospitals specializing in pulmonary diseases, with high experienced technics and the sophisticated laboratory conditions. We simultaneously compared T-SPOT.TB and QFT-GIT on the same blood samples from the same participants for each test. This may be caused the high concordance of sensitivity between the two IGRAs. Furthermore, The QFT-GIT is based on the ELISA method, while T-SPOT.TB assay is based on the ELISPOT method. The sensitivity of QFT-GIT is strongly affected by immunosuppressed status with only a slight effect of antigenic load, whereas the sensitivity of T-SPOT.TB is strongly affected by the antigenic load with only a slight effect of immunosuppressed status^11,26^. The present study enrolledspecified patients with active TB with nonimmunosuppressed conditions, seems to reduce the differences between the blood tests might provide slightly different results in generalization. In addition, both T-SPOT.TB and QFT-GIT had imperfect sensitivities and poor negative predictive values (70.3% and 68.6) could give rise to potential problems when using negative results as a “rule-out” criterion in the diagnosis of active TB. Previous studies have interpreted multiple factors that may affect IGRAs^15,27,28^. Careful interpretation of negative IGRA results is necessary for patients who are older, over-weight, have HIV coinfection or are receiving TB treatment. Caution is also needed in interpretation of results based on the patient’s immunosuppression status and certain HLA-genotypes.

The diagnosis of TB in patients with negative bacteriological results remains a problem in clinical settings. In our subgroups, the sensitivities of both tests in confirmed TB and probable TB groups were not significantly different. When combining the two tests, the overall sensitivity rose to 87.5% with minimal loss of specificity (from 60.5 to 58.5%) in the probable TB group. Thus, the commercially available versions of the T-SPOT.TB and QFT-GIT assays may be of complementary diagnostic value for the assessment of bacteriologically negative TB^7,8,29^.

The low specificities of the two assays, 63.4% for T-SPOT.TB and 60.5% for QFT-GIT, limit their usefulness in routine clinical practice, at least in a setting highly endemic for TB such as in China^17,20,30^. As only a small minority of lymphocytes in the human body circulate in the blood, neither immunological test could accurately distinguish between untreated active TB and latent tuberculosis infection (LTBI) when IGRAs were performed on the blood cells^31^. Latent TB has a high prevalence, about 19% in rural China based on the QFT-GIT assay and likely contributes a decrease in the specificity of the IGRAs^32^. In this study, specificities of the tests were poor for active TB compared with previously reported data^7,8^. This study was designed to evaluate the diagnostic validity of T-SPOT.TB and QFT-GIT in routine clinical practice, and thus focused on unselected patients with suspected active TB. Therefore, in this setting, the diagnostic validity tends to be lower than that in studies in which healthy people are enrolled as negative controls and patients with active TB as positive controls. Furthermore, although the IGRAs have been confirmed to be more sensitive and specific than the tuberculin skin test (TST) since the assays use M. tuberculosis specific antigens, we should not ignore the fact that patients infected with *M. kansasii, M. szulgaior M. marinum* may also lead to positive results in IGRAs^20,28,33^.

One disadvantage of interferon-γ assays is that they can yield ITRs. Previous studies found that ITRs were common (ranging from 0% to 20%) when using QFT-GIT assay^14,15,26,28,34^. However, the majority of them were associated with immunosuppression or old age. In this study, the rate of ITRs was significantly higher for QFT-GIT than that for T-SPOT.TB. Of the 32 patients with indeterminate QFT-GIT results, only 3 showed indeterminate results by T-SPOT.TB test. Furthermore, most indeterminate results of both tests were caused by insufficient response to the positive control antigens. It can be speculated that the patients with weak immune system response to the positive control yield a response that is insufficiently strong to be measured^11,13^. The main differences between the QFT-GIT and T-SPOT.TB lie in the specimens used (whole blood versus mononuclear cells). The use of whole blood by the QFT-GIT may lead to the presence of insufficient mononuclear cells producing various kinds of cytokines, so that indeterminate QFT-GIT result was more common than for T-SPOT.TB. As for clinical characteristics, patients showing indeterminate results on both tests were most frequently elderly patients or patients with severe underlying diseases such as diabetes mellitus. In our study, the number of indeterminate results was small and there were no patients who were serum positive for HIV. As there are no reports demonstrating a decrease in the functional activity of mononuclear cells for these patients suspected of TB disease, it will be necessary to investigate the functional activity of mononuclear cells of these patients in future^15,26,34^.

Although both the T-SPOT.TB and QFT-GIT assays measure T-cell interferon-γ responses to similar M. tuberculosis-specific antigens over a 16- to 24-h incubation period, they are based on different technology platforms. The mycobacterial antigen load (which reflects bacterial load) is positively correlated with effector T cell levels^17,35^. Similar to previous studies, in the present study, the level of interferon-γ release (SFCs by T-SPOT.TB and interferon-γ concentration by QFT-GIT) from TB samples were much higher than in the non-TB patients (P < 0.001). Due to the lower interferon-γ responses, probable TB samples may have much lower antigen load than confirmed TB, but the difference did not reach statistical significance (P > 0.05). In this study, the concordance between the tests (QFT-G IT and T- SPOT.TB) using the Kappa index, was excellent (k = 0.82) similar to that in other reports^8,13,36,37^. However, when evaluating the association between the numbers of SFCs in the T-SPOT.TB and the amounts of released interferon-γ measured in the QFT-G-IT by the regression analysis, we obtained a poor correlation (r^2^ = 0.086) between the two assays (Fig. 3). A few factors should be considered to interpret this poor correlation. T-SPOT.TB requires a specific number of peripheral blood mononuclear cells and measures the number of interferon-γ-secreting T cells via ELISPOT assay, whereas QFT-GIT measures the concentration of interferon-γ via an ELISA using specific volume of whole blood. These indicate that we cannot rule out the possibility that a small number of T cells can produce a great deal of interferon-γ in some patients. ELISA (measured as “IU/mL”) is more vulnerable to the influence of incubation time than ELISPOT (measured as “SFCs/2.5 × 105 PBMCs”). In addition, the T-SPOT.TB method use of separate mixtures of ESAT-6 and CFP-10 synthetic peptides as M. tuberculosis-specific antigens, compared to the QFT-GIT use of a single mixture of synthetic ESAT-6, CFP-10, and TB7.7 peptides could also lead to the poor correlation. Previous studies have reported that the use of both techniques simultaneously can contribute to improving the knowledge of TB immunity^26,38,39^. Therefore, the results of poor correlation require further investigation to elucidate possible clinical implications.

To our best knowledge, this is the largest study to date to directly compare the two IGRAs currently commercially available for the diagnosis of active TB. However, we did not enroll LTBI subjects in the control group, which might have some impact on specificity, since the two IGRA tests cannot distinguish between LTBI and active TB. In addition, immunodeficient patients should be included to finalizing the study results.

Furthermore, QuantiFERON-TB Plus (QFT-Plus) as an updated version of QFT-GIT has been on the market in Europe and the United States, but not commercially available in China. QFT-Plus includes two tubes, TB1 and TB2 with MTB antigens to elicit a specific immune response. TB1 is designed to induce a specific CD4 T-cell response. TB2 contains newly designed peptides stimulating interferon-γ production by both CD4 and CD8 T cells^40^. The additional peptides for eliciting CD8 T-cell responses may reduce the indeterminate results of diagnosing active TB.

In conclusion, while there were some differences in the performance of the T-SPOT.TB and QFT-GIT assays, the two commercialized IGRAs have similar sensitivities to aid in the diagnosis of active TB. When combined, the two assays may be of complementary diagnostic value for probable TB. However, the high false positive rates of these tests limit their usefulness in routine clinical practice in China, where the prevalence of LTBI is high. Therefore, these assays should not be used alone to rule out or rule in active TB cases, and further modification is needed to improve their accuracy.

## Methods

### Patients and setting

This multicenter, prospective comparison study was conducted at Beijing Chest Hospital, Shanghai Pulmonary Hospital, Inner Mongolia Autonomous Region Fourth Hospital and Shenyang Chest Hospital in China. The four hospitals are situated in the south, north, east, and center of China and specialize in the diagnosis and treatment of pulmonary diseases, especially TB and lung cancer. Between June 2012 and November 2013, a total of 830 patients with presumed pulmonary TB who underwent valid T-SPOT.TB and QFT-GIT tests before the onset of anti-TB therapy were enrolled in the study. Patients gave written informed consent and were included if they had any clinical symptoms, signs or radiographic evidence of active TB. Patients were excluded if they had a history of previous TB or TB contact or had received anti-TB therapy before enrollment.

Medical records were collected on age, gender, underlying disease, HIV serology and duration of illness before hospitalization. The ‘duration of illness before hospitalization’ was defined as the length of time from the onset of TB clinical symptoms to the time of admission to hospital. Routine clinical, microbiologic, histopathological, radiological examinations were also performed and collected over a follow-up period of at least 3 months. The microbiologic examinations included, at a minimum, three sputum smears and one sputum culture. All patients were interviewed and a questionnaire was completed to obtain epidemiological data.

This study was performed in accordance with the guidelines of the Helsinki Declaration and was approved by the Ethics Committee of the Beijing Chest Hospital, Capital Medical University.

### Definitions and diagnosis

All participants were classified into one of 3 groups depending on their clinical manifestations, bacteriological, biochemical examinations, histopathological examination and responses to anti-TB therapies. The first group, designated as confirmed TB, consisted of patients with microbiologically confirmed pulmonary TB who tested positive three 3 times in sputum smears or culture analyses. The second group was designated as probable TB, consisting of patients who were classified as having pulmonary TB based on clinical and radiological findings, but lacking microbiological evidence of *M. tuberculosis* infection. All patients with confirmed TB or probable TB responded clinically to antituberculous chemotherapy during follow-up and were categorized as having active TB^41^. The final group, designated as the control group (non-TB), contains patients categorized as not having active TB if other diagnoses were made or if clinical improvement occurred without recent anti-TB therapy.

### T-SPOT.TB assay

The T-SPOT.TB (Oxford Immunotec Limited, UK) was performed according to the manufacturer’s instructions in Class II Biological Safety Cabinet (ESCO A2, Singapore). Six mL of heparinized peripheral blood samples were collected and processed within 2 h of collection. Peripheral blood mononuclear cells (PBMCs) were isolated and adjusted to a concentration of 2.5 × 10^6^/mL and then the wells were stimulated with 50 μL each of phytohemagglutinin (positive control), secretory antigenic target (ESAT)-6, culture filtrate protein (CFP)-10, and AIM® V medium (Invitrogen, USA) (negative control). The procedure was performed in plates pre-coated with anti-interferon-γ antibodies at 37 °C for 16 to 20 hours. After application of the alkaline phosphatase-conjugated second antibody and chromogenic substrate, the number of spot forming cells (SFCs) in each well was automatically counted with a CTL ELISPOT system (CTL- ImmunoSpot® S5 Versa Analyzer, USA). The criteria for positive, negative, and indeterminate outcomes were recommended by the manufacturer^33^.

### QuantiFERON-TB Gold In-Tube assay

The QFT-GIT (Cellestis Limited, Australia) was performed according to the manufacturer’s instructions in Class II Biological Safety Cabinet (ESCO A2, Singapore). Three mL peripheral blood was drawn from each patient on the day of enrollment. The blood was collected in 3 special tubes: 1 coated with *M. tuberculosis* -specific peptides (TBAg: ESAT-6, CFP-10, and TB 7.7), 1 coated with mitogen as a positive control, and 1 without antigen coating as a negative control (Nil). Within 8 h of blood sampling, the tubes were incubated for 16–24 h at 37 °C, centrifuged, and stored at −20 °C until assayed (within 7 days). The plasma interferon-γ concentration was measured by ELISA. The test results were determined as negative, indeterminate, or positive (cutoff at 0.35 IU/mL) according to the manufacturer’s software.

### Statistical analysis

Data analysis was performed using SPSS, Version 17.0 (SPSS, Inc, Chicago, IL, USA). Categorical data were compared by Pearson’s Chi-square or Fisher’s exact test. Odds ratio (OR) and multiple logistic regression analysis were used to calculate crude and adjusted risk, and model building was performed backward using the chance criteria for variable selection. Covariates that were significant in bivariate analyses were included in the preliminary model. Other covariates that were considered biologically important were forced into the model irrespective of statistical significance. In addition, sensitivity, specificity, positive predictive value (PPV), negative predictive value (NPV), likelihood ratio positive (LR+), and likelihood ratio negative (LR−) were calculated to evaluate diagnostic performance for the T-SPOT.TB and QFT-GIT assays. Ninety five percent confidence intervals (95% CI) were estimated according to the binomial distribution. Continuous variables were compared using nonparametric Mann-Whitney U test. All *P* values reported were calculated two-tailed with statistical significance set to *P* < 0.05.

## References

[1] World Health Organization. Global tuberculosis control. 2016. Available at: http://www.who.int/tb/publications/global_report/en/ (2016).

[2] Wang L, Liu J, Chin DP (2007). Progress in tuberculosis control and the evolving public-health system in China. Lancet..

[3] Li Y (2013). Factors associated with patient, and diagnostic delays in Chinese TB patients: a systematic review and meta-analysis. BMC. Med..

[4] Brodie D, Schluger NW (2005). The diagnosis of tuberculosis. Clin. Chest. Med.

[5] Lalvani A, Pareek M (2010). Interferon gamma release assays: principles and practice. Enferm. Infecc. Microbiol. Clin..

[6] Mazurek GH (2010). IGRA Expert Committee; Centers for Disease Control and Prevention (CDC). Updated guidelines for using interferon gamma release assays to detect Mycobacterium tuberculosis infection - United States, 2010. MMWR. Recomm. Rep..

[7] Zhang S (2010). Evaluation of gamma interferon release assays using Mycobacterium tuberculosis antigens for diagnosis of latent and active tuberculosis in Mycobacterium bovis BCG-vaccinated populations. Clin. Vaccine. Immunol..

[8] Xia H (2015). Diagnostic values of the QuantiFERON-TB Gold In-tube assay carried out in China for diagnosing pulmonary tuberculosis. PLoS One.

[9] Ferrara G (2006). Use in routine clinical practice of two commercial blood tests for diagnosis of infection with Mycobacterium tuberculosis: a prospective study. Lancet..

[10] Metcalfe JZ (2011). Interferon-gamma release assays for active pulmonary tuberculosis diagnosis in adults in low- and middle-income countries: systematic review and meta-analysis. J. Infect. Dis..

[11] Hong SI, Lee YM, Park KH, Kim SH (2011). Is the sensitivity of the QuantiFERON-TB gold in-tube test lower than that of T-SPOT.TB in patients with miliary tuberculosis?. Clin. Infect. Dis..

[12] Altet-Gomez N (2011). Diagnosing TB infection in children: analysis of discordances using *in vitro* tests and the tuberculin skin test. Eur.Respir.J..

[13] Aggarwal AN, Agarwal R, Gupta D, Dhooria S, Behera D (2015). Interferon Gamma Release Assays for Diagnosis of Pleural Tuberculosis: a Systematic Review and Meta-Analysis. J.Clin. Microbiol..

[14] Beffa P, Zellweger A, Janssens JP, Wrighton-Smith P, Zellweger JP (2008). Indeterminate test results of T-SPOT.TB performed under routine field conditions. Eur. Respir. J..

[15] Cho K (2012). Factors Associated with Indeterminate and False Negative Results of QuantiFERON-TB Gold In-Tube Test in Active Tuberculosis. Tuberc, Respir. Dis (Seoul)..

[16] Pai M, Zwerling A, Menzies D (2008). Systematic review: T-cell-based assays for the diagnosis of latent tuberculosis infection: an update. Ann. Intern. Med..

[17] Diel R, Loddenkemper R, Nienhaus A (2010). Evidence-based comparison of commercial interferon-gamma release assays for detecting active TB: a metaanalysis. Chest..

[18] Sester M (2011). Interferon-gamma release assays for the diagnosis of active tuberculosis: a systematic review and meta-analysis. Eur. Respir. J..

[19] Kobashi Y (2008). Usefulness of the QuantiFERON TB-2G test for the differential diagnosis of pulmonary tuberculosis. Intern. Med..

[20] Lu P, Chen X, Zhu LM, Yang HT (2008). Interferon-Gamma Release Assays for the Diagnosis of Tuberculosis: A Systematic Review and Meta-analysis. Lung..

[21] Feng Y (2012). Interferon-gamma release assay performance in pulmonary and extrapulmonary tuberculosis. PLoS One.

[22] Kang W (2018). Interferon-Gamma Release Assay is Not Appropriate for the Diagnosis of Active Tuberculosis in High-Burden Tuberculosis Settings: A Retrospective Multicenter Investigation. Chin. Med. J (Engl)..

[23] Dai Y (2012). Evaluation of interferon-gamma release assays for the diagnosis of tuberculosis:An updated meta-analysis. Eur. J. Clin. Microbiol. Infect. Dis..

[24] Michael JS (2008). Comparison of QuantiFERON-TB Gold In-Tube to Tuberculin Skin Test for the Diagnosis of Active Tuberculosis (TB) in India - Preliminary Analysis. Int. J. Infect. Dis..

[25] Adetifa IM (2007). Comparison of two interferon gamma release assays in the diagnosis of Mycobacterium tuberculosis infection and disease in the Gambia. BMC.Infect.Dis..

[26] Bae W (2016). Comparison of the Sensitivity of QuantiFERON-TB Gold In-Tube and T-SPOT.TB According to Patient Age. PLoS One.

[27] Lee YM (2013). Risk factors for false-negative results of T-SPOT.TB and tuberculin skin test in extrapulmonary tuberculosis. Infection..

[28] Pan L (2015). Risk factors for false-negative T-SPOT.TB assay results in patients with pulmonary and extra-pulmonary TB. J. Infect..

[29] Hermansen TS, Thomsen VO, Lillebaek T, Ravn P (2014). Non-tuberculous mycobacteria and the performance of interferon gamma release assays in Denmark. PLoS One.

[30] Ling DI (2013). Incremental value of T-SPOT.TB for diagnosis of active pulmonary tuberculosis in children in a high-burden setting: a multivariable analysis. Thorax..

[31] Jafari C (2006). Rapid diagnosis of smear-negative tuberculosis by bronchoalveolar lavage enzyme-linked immunospot. Am. J. Respir. Crit. Care. Med..

[32] Gao L (2015). Latent tuberculosis infection in rural China: baseline results of a population-based, multicentre, prospective cohort study. Lancet. Infect. Dis..

[33] Oxford Immunotec Limited.T-SPOT. TB Accessed 20 February 2011; Available from: Available at: http://www.oxfordimmunotec.com/T-SPOT_International (2011).

[34] Lange B, Vavra M, Kern WV, Wagner D (2010). Indeterminate results of a tuberculosis-specific interferon-gamma release assay in immunocompromised patients. Eur. Respir. J..

[35] Nicol MP (2005). Enzyme-linked immunospot assay responses to early secretory antigenic target 6, culture filtrate protein 10, and purified protein derivative among children with tuberculosis: implications for diagnosis and monitoring of therapy. Clin. Infect. Dis..

[36] Chee CB (2008). Comparison of sensitivities of two commercial gamma interferon release assays for pulmonary tuberculosis. J. Clin. Microbiol..

[37] Gan SH (2014). Interferon-gamma responses to Mycobacterium tuberculosis-specific antigens in diabetes mellitus. Eur. Respir. J..

[38] Komiya K (2010). Impact of peripheral lymphocyte count on the sensitivity of 2 IFN-gamma release assays, QFT-G and ELISPOT, in patients with pulmonary tuberculosis. Intern. Med..

[39] van Zyl-Smit RN, Lehloenya RJ, Meldau R, Dheda K (2016). Impact of correcting the lymphocyte count to improve the sensitivity of TB antigen-specific peripheral blood-based quantitative T cell assays (T-SPOT.((R))TB and QFT-GIT). J.Thorac.Dis..

[40] Yi L (2016). Evaluation of QuantiFERON-TB Gold Plus for Detection of Mycobacterium tuberculosis infection in Japan. Sci. Rep..

[41] China Medical Association of Tuberculosis (2001). Guideline of diagnosis and treatment on tuberculosis. Zhong. Hua. Jie. He. He. Hu. Xi. Za. Zhi..

